# A Method for Identification and Assessment of Radioxenon Plumes by Absorption in Polycarbonates

**DOI:** 10.3390/s21238107

**Published:** 2021-12-03

**Authors:** Dobromir Pressyanov, Pavel Stavrev

**Affiliations:** Faculty of Physics, University of Sofia “St. Kliment Ohridski”, 5 James Bourchier Blvd., 1164 Sofia, Bulgaria; pstavrev@phys.uni-sofia.bg

**Keywords:** radioxenon plumes, ^133^Xe, absorption in polycarbonates, Makrofol, retrospective estimates, nuclear accidents

## Abstract

A method for the retrospective evaluation of the integrated activity concentration of ^133^Xe during radioxenon plumes and the moment of the plume’s center is proposed and explored by computer modeling. The concept is to use a specimen of polycarbonate material (a stack of Makrofol N foils of thickness 120 µm and 40 µm in 1 L non-hermetic Marinelly beaker) that is placed in the environment or in a controlled nuclear or radiopharmaceutical facility. On a regular basis or incidentally, the specimen may be retrieved and gamma spectrometry in two consecutive time intervals with durations of 8 h and 16 h is performed. To assess the performance of the method, ^133^Xe plumes of various integrated activity concentrations and with a duration of up to 10 h are simulated and analyzed, assuming that the measurement starts with a delay of up to one day after the moment of the plume center. It is found that the deviation between the estimates by the method and their true values are within a few percent. Depending on the delay, events of integrated ^133^Xe activity concentration 250–1000 Bq h m^−3^ might be qualitatively identified. At levels >10,000 Bq h m^−3^, the uncertainty of the quantitative estimates might be ≤10%.

## 1. Introduction

Among the man-made radioactive noble gases, the isotopes of xenon (radioxenon, isotopes of interest are ^135^Xe, ^133^Xe, ^133m^Xe and ^131m^Xe) attract particular attention. These isotopes are released from nuclear and radiopharmaceutical facilities and from hospitals, where ^133^Xe is administered to patients. They are among the key radionuclides whose release from nuclear power plants (NPPs) has to be monitored [1]. In the event of a nuclear emergency, most of their inventory in the nuclear installation may be released into the environment [2]. Radioxenon plays a key role in the control of the Treaty on the Non-Proliferation of Nuclear Weapons and the Comprehensive Test Ban Treaty (CTBT) and a world monitoring network has been set up for this purpose [3]. In the environment, the concentrations of ^133^Xe usually exceed those of other xenon isotopes by several orders of magnitude [4], but, after a subsurface nuclear explosion, the ratio of ^135^Xe/^133^Xe may be four orders of magnitude larger compared, for instance, to a reactor release [5]. The estimated release of ^133^Xe from NPPs in North America and Europe ranges within 10^7^–10^11^ Bq/day per one NPP unit [6], mostly as continuous emissions. The emissions from radiopharmaceutical factories are substantially higher [7] and, usually, these are “pulse” short-term releases, reaching activity of 10^15^ Bq in several hours, in some cases [4]. The ^133^Xe release after the Chernobil accident was estimated to be between 1.33 × 10^17^ and 6.5 × 10^18^ Bq [8] and the ^133^Xe release after the Fukushima Daiichi accident in 2011 was estimated to be of the order of 10^19^ Bq [9]. For comparison, after a 1 kt nuclear explosion of plutonium A-bomb, approximately 10^15^ Bq of ^133^Xe will be emitted within three hours of the explosion [10,11].

Continuous reactor releases are hardly detectable at long distances from NPPs. In contrast, radioxenon plumes with a duration of several hours and integrated over the duration of the plume ^133^Xe activity concentration of more than 1 kBq h m^−3^ may be observed tens of kilometers away from the radiopharmaceutical facilities after short-term releases of ^133^Xe of the order of 10^13^–10^14^ Bq [4]. At such distances, the plumes after a major nuclear emergency can be expected to have an integrated activity concentration that is 3–5 orders of magnitude greater. They are also detected at much longer distances: after the Fukushima Daiichi accident, ^133^Xe activity concentrations of up to 30–70 Bq m^−3^ were detected at distances >6500 km from the site of emergency [12,13]. The plumes at longer distances also last longer due to the plume dispersion during atmospheric transport.

Measuring ^133^Xe in the environment is a challenge. Thus far, there are no compact and mobile monitors that are sufficiently sensitive to be used for measurement in the environment around nuclear and radiopharmaceutical facilities. The sensitivity of the stationary monitors used in nuclear installation is not sufficient for environmental control, and, in the case of a major nuclear emergency, they may become inoperative. The radioxenon detection systems used in CTBT stations are highly sensitive, but, after a major nuclear emergency, they may reach their upper limit of detection and become inoperative. On the other hand, strategic decisions rely on information about ^133^Xe in the environment, especially after a nuclear emergency, accidental release from nuclear and radiopharmaceutical facilities or in the case of possible clandestine nuclear actions.

The use of the high absorption ability of noble gases by bisphenol-A based polycarbonates (BPA-PCs) [14] for ^222^Rn measurements was first proposed in 1999 [15] and for the measurements of ^85^Kr and ^133^Xe in 2004 [16]. Since then, many studies have focused on the further development of this “polycarbonate method” for natural [17] and man-made radioactive noble gases [18,19,20,21]. In particular, different BPA-PCs have been studied and Makrofol N^®^ (Bayer AG) has been identified as the material that has the highest absorption ability for noble gases [22,23,24]. Thus far, the potential of this method for ^133^Xe measurement has been studied for long-term (days/weeks) continuous exposures [19,21]. However, accidental radioxenon releases, as well as those from radiopharmaceutical facilities, are mostly short-term “pulses”, which are registered in the close environment as radioxenon plumes with a duration of several hours [4].

In this work, we propose a method to identify, qualitatively, ^133^Xe plumes and to evaluate, quantitatively, the integrated ^133^Xe activity concentration and the moment of the plume center. The method consists of placing in the environment or within the controlled facility of polycarbonate specimens formed as a stack of Makrofol N foils of two different thicknesses, which are placed in a non-hermetic canister. The specimens can be left for a long time and taken for analysis only in the case of suspected high radioxenon release or on a regular basis, e.g., daily, for performing regular control. Then, the specimens are transported to a laboratory, which may be distant from the point of exposure, and measured via HPGe gamma spectrometry, using the 81 keV line of ^133^Xe. By measuring the signal (net area of the 81 keV peak spectral region of interest) in two different time intervals, the integrated activity concentration of ^133^Xe during the plume and the time (before the start of the measurement) of the “center of the plume” can be evaluated. By computer modeling of realistic situations, it is demonstrated that the method provides feasible results and is sufficiently sensitive in the range that is frequently encountered in the environment, e.g., around radiopharmaceutical factories [4]. The specimen can be analyzed up to one day after the plume has dissipated.

## 2. Materials and Methods

### 2.1. The Concept and Basics of the Method

**Figure 1:**
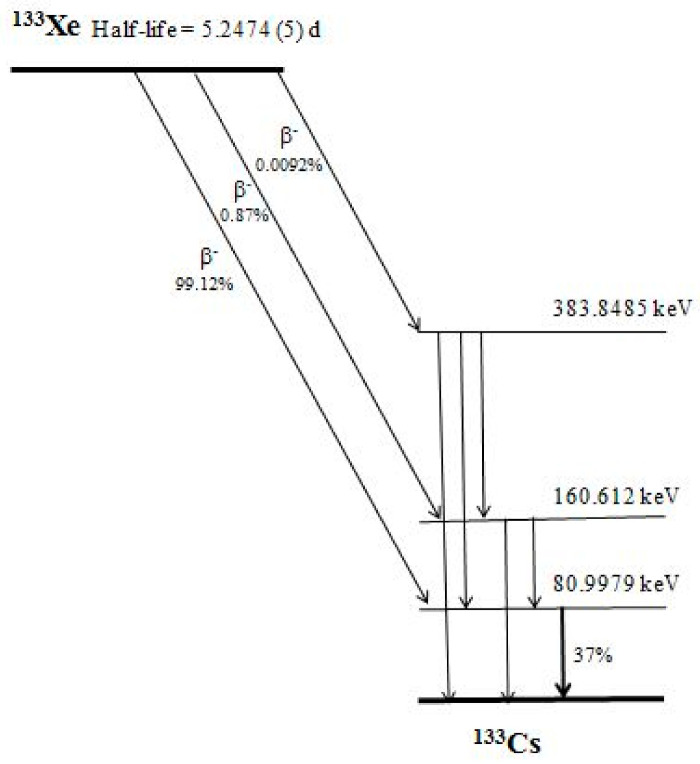
Decay scheme of ^133^Xe. The half-life, probability of the decay modes and energy of the excited states are shown, using the data of Ref. [25]. The present method uses the gamma-line of energy 80.9972 (≈81 kev) that is of emission probability of 37%.

The decay scheme and the basic nuclear data of the isotope ^133^Xe are shown in Figure 1 [25].

**Figure 2:**
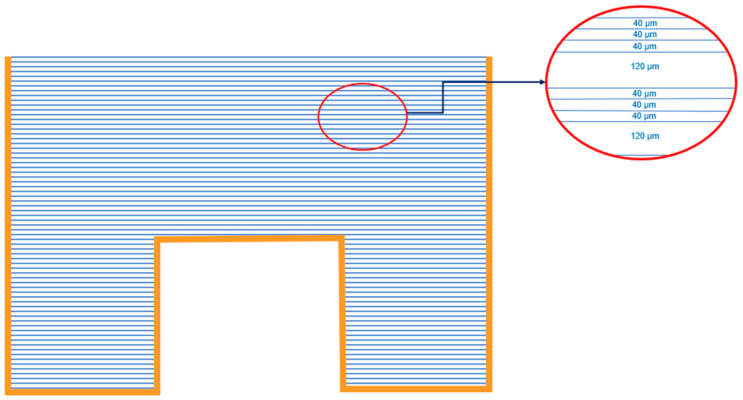
Placement of Makrofol N foils in 1 L Marinelli beaker for exposure in the environment. Above one 120 μm foil, three 40 μm foils are placed and this is repeated until the volume is filled.

Here, we employ the concept of a “composite absorber”, consisting of a stack of absorbing foils. When the foils are not hermetically stuck together, the noble gas may penetrate freely between them. Initially, this concept was proposed and tested for radon measurement [22,26,27,28,29] and further evaluated for ^133^Xe, too [19]. Previous experiments with ^222^Rn have shown good agreement between the theoretical modeling of such “composite absorbers” and experimental results [29]. For simulation purposes, the following experimental setup is assumed, namely Makrofol N foils of thickness 40 μm and 120 μm, stuck together and placed in a canister, specifically a 1 L Marinelli beaker, as shown in Figure 2. Half of the volume of the beaker is filled with 40 μm and half of it with 120 μm, and the foils of each type can be considered evenly distributed within the volume.

The transport of a radioactive isotope of a noble gas in a polycarbonate is described by the diffusion equation with radioactive decay [18,21,30]. For the geometry of thin foils of thickness *L*, it is given by Equation (1) [30]:

(1) $$\frac{\partial n}{\partial t}=D\frac{\partial^{2}n}{\partial x^{2}}-\lambda n$$

where *n* is the atomic concentration of the noble gas in the polycarbonate material, *x* = 0 and *x* = *L* are the coordinates of the foil boundaries, *D* is the diffusion coefficient of xenon in the polycarbonate material, and *λ* is the decay constant of the isotope (in the present case, this is ^133^Xe). During exposure, the initial and boundary conditions are *n*(*x, t* = 0) = 0 and *n*(*x* = 0, *t*) = *n*(*x* = *L*, t) = *Kn*_0_*(t)*, where *n*_0_ is the atom concentration of the noble gas in the ambient air and *K* is the “partition coefficient” (dimensionless solubility) of the gas in the material (*K* = ratio of the concentration in an “infinitely” thin surface layer of the material to that in the air). After exposure, the boundary conditions are zero and the initial distribution of *n* within the foil is that at the end of the preceding exposure. This model was found to perfectly fit the experimental data [18,21,24,30].

The exact solutions of Equation (1) have been obtained elsewhere [18,30]. According to these solutions, during exposure to a constant activity concentration *c_A_* in air (*c_A_* = *λn_0_*), the growth in activity in a stack of foils of thickness *L* and of total volume *V* can be expressed as:

(2) $$A\left(t\right)\;=\underset{V}{\int }\lambda n\left(x,t\right)dV=c_{A}V\left\{\frac{8\lambda KL_{D}^{2}}{L^{2}}\overset{\infty }{\underset{k=0}{\sum }}\frac{1-exp\left(-\lambda_{2k+1}t\right)}{\lambda_{2k+1}}\right\}$$

where *L_D_* is the diffusion length of the considered isotope in the material ($L_{D}=\sqrt{D/\lambda }$) and the constants λ_2k+1_ are determined as follows:

(3) $$\lambda_{2k+1}=\lambda \left(1+{\left(\left(2k+1\right)\pi \frac{L_{D}}{L}\right)}^{2}\right)$$

Here, values of *K* = 17.2 and *L_D_* (^133^Xe) = 87.8 μm were used, according to data in Ref. [24]. In Ref. [24], *L_D_* was reported for ^131m^Xe, and, as the diffusion coefficient for all Xe isotopes is the same, it was recalculated for ^133^Xe as follows:

(4) LDX133e =LDX131meλX131me/λX133e

After the end of exposure at moment *T*, the activity decreases due to the radioactive decay and outgazing. The process is described by expression (5) [30]:

(5) $$A\left(t\right)\;=c_{A}V\left\{\frac{8\lambda KL_{D}^{2}}{L^{2}}\overset{\infty }{\underset{k=0}{\sum }}\frac{1-exp\left(-\lambda_{2k+1\;}T\right)}{\lambda_{2k+1\;}}exp\left(-\lambda_{2k+1\;}t\right)\right\}$$

where *t* is the time after the end of exposure. Notably, the thinner the foil, the faster is the decrease in the activity [19].

**Figure 3:**
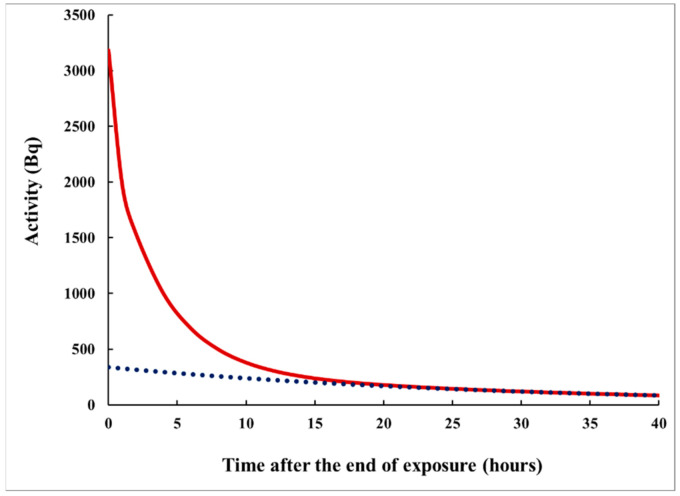
The decrease in the activity in the specimen (due to radioactive decay and outgazing) after the end of exposure, according to eqn. (6). The initial activity of the specimen (at the end of exposure) is 3200 Bq. The dotted line corresponds to the “slowest component” in the sum (6), that is ~exp($-\lambda_{1}^{\left(2\right)}T$). Note that the ratio *A*(*τ*)/*A*(*t+τ*) at fixed *t* depends on *τ*; therefore, *τ* might be correlated with *S*_1_/*S*_2_.

Let *A*_1_(*t*) be the activity, according to Equation (5), of a volume *V* filled with foils of *L* = *L*_1_ (in the present case, *L*_1_ = 40 µm) and *A*_2_(*t*) is when it is filled with foils of *L* = *L*_2_ (*L*_2_ = 120 µm). Then, the activity of the composite absorber of volume *V*, when $\frac{1}{2}$*V* is filled with foils of thickness *L*_1_ and another $\frac{1}{2}$*V* with foils of *L*_2_, is given by Equation (6):

(6) $$A\left(t\right)\;=\frac{1}{2}\left(A_{1}\left(t\right)\;+\;A_{2}\left(t\right)\right)\;=c_{A}V\left\{4\lambda K\left[\frac{L_{D}^{2}}{L_{1}^{2}}\overset{\infty }{\underset{k=0}{\sum }}\frac{1-exp\left(-\lambda_{2k+1}^{\left(1\right)}T\right)}{\lambda_{2k+1}^{\left(1\right)}}exp\left(-\lambda_{2k+1}^{\left(1\right)}t\right)+\frac{L_{D}^{2}}{L_{2}^{2}}\overset{\infty }{\underset{k=0}{\sum }}\frac{1-exp\left(-\lambda_{2k+1}^{\left(2\right)}T\right)}{\lambda_{2k+1}^{\left(2\right)}}exp\left(-\lambda_{2k+1}^{\left(2\right)}t\right)\right]\right\}$$

where the constants $\lambda_{2k+1}^{\left(i\right)}$ correspond to foils of thickness *L_i_* (*i* = 1,2). Figure 3 illustrates the time dependence of *A*(*t*).

When using HPGe gamma spectrometry, the measured signal (*S*) is the net-area of the 81 keV peak region of interest (ROI). In data processing, the detection efficiency (*ε*) and the probability (*p*) for 81 keV gamma emission in the ^133^Xe decay should be taken into account. For modeling purposes, it is assumed that the specimens taken for analysis are measured by HPGe gamma spectrometry in two consecutive time intervals: the first of duration *t*_1_ = 8 h (signal *S*_1_) and the second of duration *t_2_* = 16 h (*S*_2_)—the duration of the whole measurement is *t*_1_ + *t*_2_ = 24 h. In the general case, the time interval when the plume has occurred is not known and some time for transportation of the specimen to the measuring laboratory may be needed. Therefore, the measurement starts with some delay *τ_d_* after the end of the plume (or τ after its “center”, for rectangular plumes of duration *T*: *τ* = *τ_d_* + *T/2*). Therefore, the modeled signals *S*_1_ and *S*_2_ in the first and the second interval are as follows:

(7) $$\begin{array}{c} S_{1}=\varepsilon p\int_{t_{d}}^{\tau_{d}+t_{1}}A\left(t\right)dt=\varepsilon pc_{A}V\{4\lambda K[\frac{L_{D}^{2}}{L_{1}^{2}}\sum_{k=0}^{\infty }\frac{1-exp\left(-\lambda_{2k+1}^{\left(1\right)}T\right)}{\lambda_{2k+1}^{\left(1\right)}\lambda_{2k+1}^{\left(1\right)}}exp\left(-\lambda_{2k+1}^{\left(1\right)}\tau_{d}\right)\left[1-exp\left(-\lambda_{2k+1}^{\left(1\right)}t_{1}\right)\right]+\\ \frac{L_{D}^{2}}{L_{2}^{2}}\sum_{k=0}^{\infty }\frac{1-exp\left(-\lambda_{2k+1}^{\left(2\right)}T\right)}{\lambda_{2k+1}^{\left(2\right)}\lambda_{2k+1}^{\left(2\right)}}exp\left(-\lambda_{2k+1}^{\left(2\right)}\tau_{d}\right)\left[1-exp\left(-\lambda_{2k+1}^{\left(2\right)}t_{1}\right)\right]]\}, \end{array}$$

where the activity *A*(*t*) is that given by Equation (6). Respectively, for *S*_2_, one obtains:

(8) $$\begin{array}{c} S_{2}=\varepsilon p\overset{\tau_{d}+t_{1}+t_{2}}{\underset{t_{d+t_{1}}}{\int }}A\left(t\right)dt\;\\ \;\;\;\;\;\;=\varepsilon pc_{A}V\{4\lambda K[\frac{L_{D}^{2}}{L_{1}^{2}}\overset{\infty }{\underset{k=0}{\sum }}\frac{1-exp\left(-\lambda_{2k+1}^{\left(1\right)}T\right)}{\lambda_{2k+1}^{\left(1\right)}\lambda_{2k+1}^{\left(1\right)}}exp\left(-\lambda_{2k+1}^{\left(1\right)}\left(\tau_{d}+t_{1}\right)\right)\left[1-exp\left(-\lambda_{2k+1}^{\left(1\right)}t_{2}\right)\right]\\ \;\;\;\;\;\;+\frac{L_{D}^{2}}{L_{2}^{2}}\overset{\infty }{\underset{k=0}{\sum }}\frac{1-exp\left(-\lambda_{2k+1}^{\left(2\right)}T\right)}{\lambda_{2k+1}^{\left(2\right)}\lambda_{2k+1}^{\left(2\right)}}exp\left(-\lambda_{2k+1}^{\left(2\right)}\left(\tau_{d}+t_{1}\right)\right)\left[1-exp\left(-\lambda_{2k+1}^{\left(2\right)}t_{2}\right)\right]]\} \end{array}$$

It could be seen in Figure 3 that, after sufficient delay (e.g., >20 h) following the end of exposure, the time dependence is determined mainly by one component of the sum (7): that with $\lambda_{1}^{\left(2\right)}$, which is the smallest constant in the set of $\lambda_{2k+1}^{\left(1,2\right)}$ constants (in present case, $\lambda_{1}^{\left(2\right)}$ = 0.0346 h^−1^). This component is also dominating in the times when *S*_2_ is acquired (*t* > 8 h). Therefore, at the times when this component dominates, the following approximation becomes useful:

(9) $$A\left(t\right)\;\approx \frac{L_{D}^{2}}{L_{2}^{2}}\frac{1-exp\left(-\lambda_{1}^{\left(2\right)}T\right)}{\lambda_{1}^{\left(2\right)}}exp\left(-\lambda_{1}^{\left(2\right)}t\right)$$

The next approximation is for a plume duration of a few hours. With the quoted *λ*_1_^(2)^, for such plumes, *λ*_1_^(2)^*T* is much smaller than one, and 1-exp($-\lambda_{1}^{\left(2\right)}T$) ≈ $\lambda_{1}^{\left(2\right)}T$**,** which, for *T* = 1 h, is valid within 2%; for *T* = 8 h, within 8%, and for *T* = 10 h, within 15%. Therefore,

(10) $$\frac{L_{D}^{2}}{L_{2}^{2}}\frac{1-exp\left(-\lambda_{1}^{\left(2\right)}T\right)}{\lambda_{1}^{\left(2\right)}}exp\left(-\lambda_{1}^{\left(2\right)}t\right)\approx \frac{L_{D}^{2}}{L_{2}^{2}}Texp\left(-\lambda_{1}^{\left(2\right)}t\right)$$

**Figure 4:**
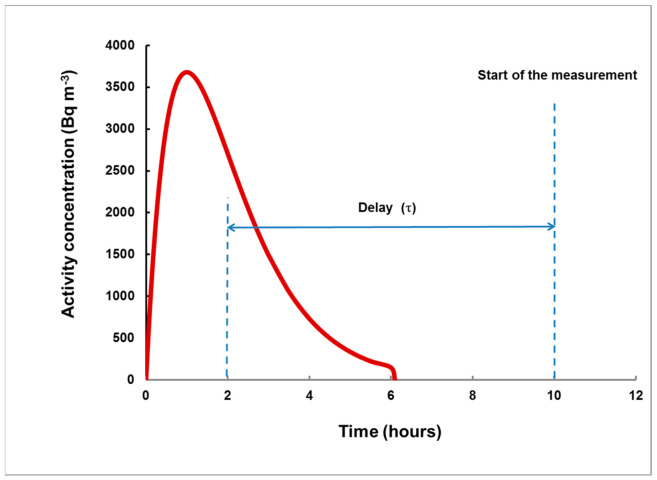
An arbitrary example of a plume. As the “end of plume” may be not well-defined, it is preferred to use the delay from the center of the plume. The moment of this center is = $\int tc_{A}dt/\int c_{A}dt$ (for rectangular plumes, it is *T*/2).

Using the approximations (7) and (8), one obtains, if the conditions of the approximations are met,

(11) $$S_{2}\approx c_{A}T\left\{\varepsilon pV\frac{4\lambda KL_{D}^{2}}{L_{2}^{2}}\frac{exp\left(-\lambda_{1}^{\left(2\right)}\left(\tau_{d}+t_{1}\right)\right)\left(1-exp\left(-\lambda_{1}^{\left(2\right)}t_{2}\right)\right)}{\lambda_{1}^{\left(2\right)}}\right\}=I_{A}\left\{\varepsilon pV\frac{4\lambda KL_{D}^{2}}{L_{2}^{2}}\frac{exp\left(-\lambda_{1}^{\left(2\right)}\left(\tau_{d}+t_{1}\right)\right)\left(1-exp\left(-\lambda_{1}^{\left(2\right)}t_{2}\right)\right)}{\lambda_{1}^{\left(2\right)}}\right\}$$

where *I_A_ = c_A_*_0_*T* is the time-integrated concentration of ^133^Xe for the considered rectangular plume. In addition, for plumes of different shapes, the end of the plume is not a well-defined moment and, preferably, the delay (*τ*) should be measured from its center, as shown in Figure 4. The following “working hypothesis”, raised for plumes of any shape and of any duration, so far not exceeding 10 h, is at the core of the method:

- For measurement in two consecutive time intervals, (0–8 h) and (8–24 h), the observed ratio *S*_1_/*S*_2_ can be correlated with the delay *τ*, measured from the center of the plume, and the dependence *τ = τ(S*_1_/*S*_2_*)* can be used to assess the delay *τ* of a plume of any shape.
- The signal *S*_2_ may be expressed as:

(12) $$S_{2}=\varepsilon p\frac{4\lambda VKL_{D}^{2}}{L_{2}^{2}}\frac{I_{A}}{\lambda_{1}^{\left(2\right)}}exp\left(-\lambda_{1}^{\left(2\right)}\left(\tau +t_{1}\right)\right)\left(1-exp\left(-\lambda_{1}^{\left(2\right)}t_{2}\right)\right)g\left(\tau \right),$$

where *g(τ)* is a “corrective function” that depends only on the delay *τ* and that accounts for the difference in (11) from the exact expression (8), which is due to the approximations made and to the replacing of *τ_d_* with *τ* for the delay.

The dependence *τ*(*S*_1_/*S*_2_) was obtained by simulation of rectangular plumes of different duration and different delay *τ*, and looking for the best fit, which can be interpolated by analytical expression. Using the same simulation data, the corrective function *g*(*τ*) was modeled by the following expression, obtained from Equation (12):

(13) $$g\left(\tau \right)\;=\frac{S_{2\;}\left(true\right)}{\varepsilon p\frac{4\lambda VKL_{D}^{2}}{L_{2}^{2}}\frac{I_{A}}{\lambda_{1}^{\left(2\right)}}exp\left(-\lambda_{1}^{\left(2\right)}\left(\tau +t_{1}\right)\right)\left(1-exp\left(-\lambda_{1}^{\left(2\right)}t_{2}\right)\right)}\;,$$

where *S*_2_(*true*) is that calculated by Equation (8).

When the above conditions are met and *τ*(*S*_1_/*S*_2_) and *g*(*τ*) are known, for any arbitrary plume, using the measured *S*_1_ and *S*_2_, the delay *τ* is determined from *τ* = *τ*(*S*_1_/*S*_2_) and *I_A_* is determined as follows:

(14) $$I_{A}=\frac{1}{\varepsilon p}\frac{L_{2}^{2}}{L_{D}^{2}}\frac{S_{2}\lambda_{1}^{\left(2\right)}}{4\lambda VK}exp\left(\lambda_{1}^{\left(2\right)}\tau \right)\frac{1}{\left(exp\left(-\lambda_{1}^{\left(2\right)}t_{1}\right)-exp\left(-\lambda_{1}^{\left(2\right)}\left(t_{1}+t_{2}\right)\right)\right)g\left(\tau \right)}$$

**Figure 5:**
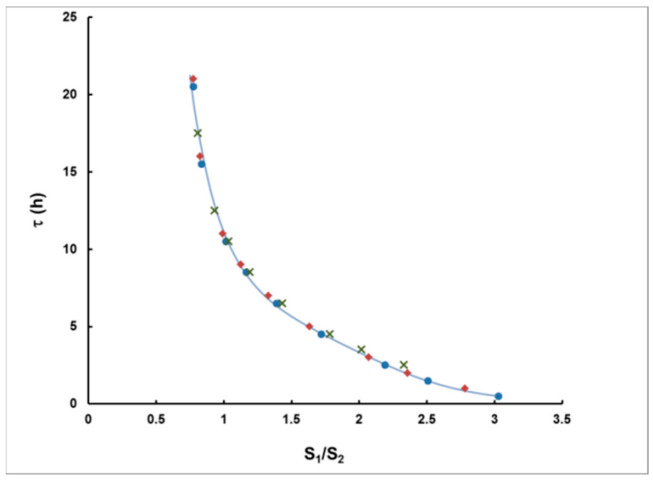
Delay (*τ*) as a function of *S*_1_/*S*_2_. Simulated rectangular plumes of duration 1 h (●), 2 h (♦) and 5 h (**×**) are fitted by the equation (15) (the solid curve).

Computer modeling, based on the analytical expressions described in this paper, was carried out in order to prove that, based on this hypothesis, one may obtain reliable results for the delay *τ* and the integrated activity concentration *I_A_*. For different *c_A_*, *T* and *τ*, the signals *S*_1_ and *S*_2_ were calculated using the exact expressions (7) and (8). The correlation between *τ* and *S*_1_/*S*_2_ is illustrated in Figure 5. The dependence is practically one and the same for rectangular plumes of duration 1–5 h.

The data are very well-interpolated by the following expression (the curve in Figure 5), which is hereafter used to determine *τ* from *S*_1_/*S*_2_:

(15) $$\tau \;\left[h\right]\;=exp\left(0.2987{\left(\frac{S_{1}}{S_{2}}\right)}^{4}-2.6469{\left(\frac{S_{1}}{S_{2}}\right)}^{3}+8.0991{\left(\frac{S_{1}}{S_{2}}\right)}^{2}-11.455\left(\frac{S_{1}}{S_{2}}\right)+8.1133\right),$$

**Figure 6:**
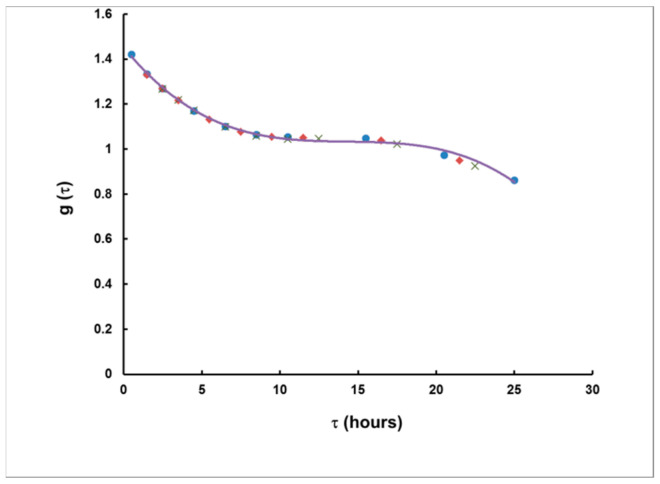
The corrective function *g* as a function of *τ*. Points correspond to rectangular plumes of duration 1 h (●), 3 h (♦) and 5 h (**×**). The solid curve represents a third-degree polynomial fit to the data points (Equation (14)).

The results for *g* are illustrated in Figure 6. As seen, the results depend on *τ*, but practically not on *T*.

This dependence was very well-interpolated analytically, by the following polynomial:

(16) $$g\left(\tau \right)\;=-0.000138\tau^{3}+0.005945\tau^{2}-0.086074\tau +1.453013,$$

where *τ* is previously obtained by Equation (15). The obtained interpolations of *τ*(*S*_1_/*S*_2_) and *g*(*τ*) were used in the data processing of simulated plumes of any shape and of different delay.

### 2.2. Estimating the Uncertainty and the Level of Plume Identification

Both *I_A_* and *τ* are functions of *S*_1_ and *S*_2_, which are uncorrelated variables. In real measurements, they are random numbers of “counting uncertainty” *σ*(*S*_1_) and *σ*(*S*_2_), which can be determined by the Poisson distribution of the total number of counts and the background. Using the interpolation expressions for *τ*(*S*_1_/*S*_2_), *g*(*τ*(*S*_1_/*S*_2_)) and replacing them in Equation (14), one obtains the function *I_A_* (*S*_1_, *S*_2_). Then, the uncertainties are calculated, using the common uncertainty propagation formulas for functions of uncorrelated variables [31]:

(17) $$\sigma \left(\tau \right)\;=\sqrt{{\left(\frac{\partial \tau }{\partial S_{1}}\right)}^{2}\sigma^{2}\left(S_{1}\right)+{\left(\frac{\partial \tau }{\partial S_{2}}\right)}^{2}\sigma^{2}\left(S_{2}\right)}$$

(18) $$\sigma \left(I_{A}\right)\;=\sqrt{{\left(\frac{\partial I_{A}}{\partial S_{1}}\right)}^{2}\sigma^{2}\left(S_{1}\right)+{\left(\frac{\partial I_{A}}{\partial S_{2}}\right)}^{2}\sigma^{2}\left(S_{2}\right)}$$

The level of plume identification *I_A_*^(*min*)^ is the minimum level at which the signal will exceed the background at 95% probability. This problem has been considered elsewhere [32], where it has been shown that, for a “well-defined background” (assumed in our modeling), this is valid when the signal is:

(19) $$S>L_{c}=1.64\sqrt{n_{b}t}$$

where *n_b_* is the “blank counting rate” (in the case of gamma spectrometry, it will be continuum+background counting rate in the region of interest of the analytical peak) and *t* is the duration of the measurement (*t = t*_1_ for *S*_1_ and *t*_2_ for *S*_2_, respectively). Simulations were carried out to obtain the integrated activity concentration *I_A_*^(*min*)^, which corresponds to the “critical level” *L_c_*. Notably, this is a level of qualitative “identification” indicating the existence of an event that resulted in a ^133^Xe signal above the background at 95% statistical significance. To have reliable quantitative estimates of *τ* and *I_A_*, the signal should be sufficiently above the “level of identification”, as shown in the Results section.

## 3. Results

### 3.1. Methodological Bias in the Estimates of the of Delay and the Integrated Concentration

**Figure 7:**
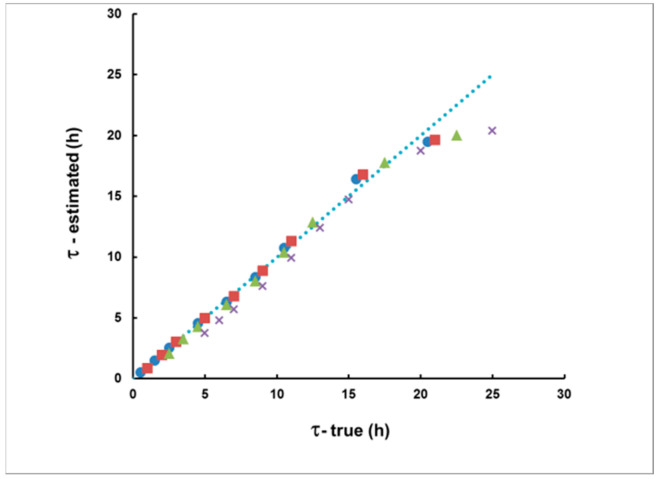
The estimated, according to S_1_/S_2_ delay, compared with the true delay of rectangular plumes of duration 1 h (●), 2 h (■), 5 h (▲) and 10 h (**×**). The dotted line represents the coincidence between the estimated and true values.

Figure 7 illustrates the agreement between the true and calculated time delay according to Equation (15). In this case, rectangular plumes with a duration of up to 10 h were considered. For time delays of up to 20 h, a very good match was observed between the true and calculated time delay values. There was a systematic bias for time delays higher than 20 h, which increased with the increase in the delay. Therefore, at this stage, the use of the proposed method should be restricted only to situations in which the measurement starts no later than 20 h after the moment of the “plume center”.

**Figure 8:**
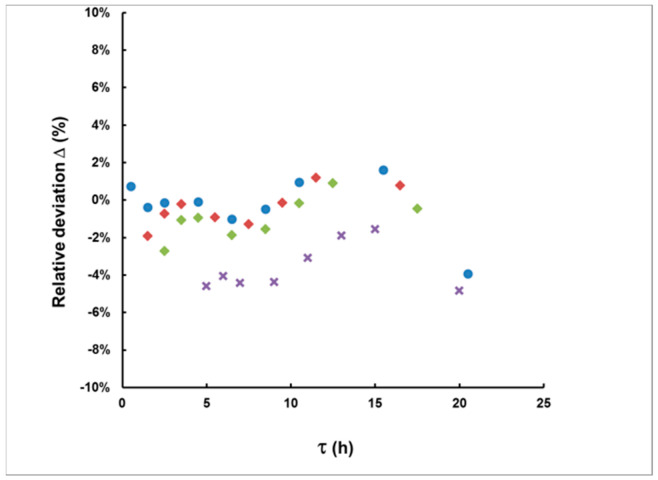
The relative deviation in *I_A_* (Δ = (*I_A_*(calculated) − *I_A_*(true))/*I_A_*(true)) for delays of up to 20 h. The duration of the simulated rectangular plumes is 1 h (●), 3 h (♦), 5 h (♦) and 10 h (**×**).

Using Equation (14) and the estimated value of the delay time, one can calculate the values of *I_A_*. Figure 8 illustrates the relative deviation of *I_A_* determined by Equation (16) from its true value for the case of rectangular plumes with a duration of up to 10 h and measurement started with a delay of up to 20 h. As seen in the figure, all relative deviations fall within 6%, while most of them fall within 2%. In most real cases, such bias will be sufficiently smaller than the uncertainty incurred by counting statistics.

### 3.2. Simulation of Levels of Identification and Instrumental Uncertainty

The characteristics of the gamma spectrometer employed for research and education, in our laboratory, were used in the modeling. The spectrometer was supplied with a HPGe detector (ORTEC^®^) of 24.9% relative efficiency and a resolution (FWHM) of 1.9 keV for the 1332 keV line of ^60^Co. Gamma spectrometry analysis uses the line of ^133^Xe with energy 81 keV (*p* = 0.37, see Figure 1) [25]. When the background (with a shielded detector) is measured for long time, the average counting rate in the region of interest of the 81 keV peak is approximately 1 cpm (60 imp/hour) and this value has been used in the modeling. Thus, the background counts in the first interval with a duration of 8 h will be 480 (8 × 60) and in the second interval with a duration of 16 h will be 960 (16 × 60) correspondingly.

**Figure 9:**
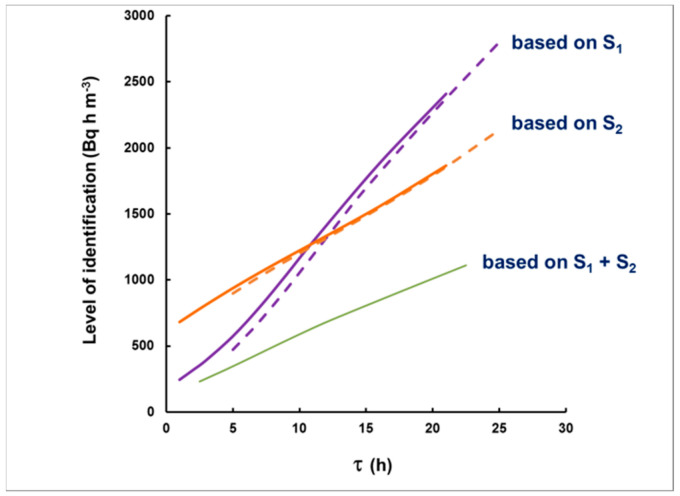
The dependence of the “level of identification” on the delay, when using *S*_1_ (violet), *S*_2_ (orange) or *S*_1_ + *S*_2_ (green). Even for *S*_1_, the dependence on plume duration is weak (dashed line). The solid lines are for plumes of duration 2 h, and the dashed lines for 10 h.

By substituting this background, one may calculate the “critical levels” *Lc_1_*, *Lc_2_*, for the corresponding intervals (see Equation (19)). The values of I_A_ that “generate” signals S_1_ = *L_C1_* or *S*_2_ = *Lc_2_* will be “the level for qualitative identification”. This level depends on the choice of the intervals used for identification and on the time delay. The results for the level of identification after numerical simulations are illustrated in Figure 9 as dependent on the delay. As seen in this figure, if *S*_1_ + *S*_2_ is used (24 h spectrum acquisition), a plume with an integrated activity concentration greater than 1000 Bq h m^−3^ will lead to a signal that would exceed the background at a 95% level of confidence, and levels as low as 250 Bq h m^−3^ may be qualitatively identified if the measurement starts with a delay <2 h. Even measurements within the first 8 h (S_1_) may be conclusive for the identification of <1000 Bq h m^−3^, provided that the delay does not exceed 10 h. This means that most of the radioxenon plumes observed, e.g., in Ref. [4], tens kilometers away from radiopharmaceutical facilities, would be identified.

However, at levels close to the level of identification, quantitative estimates will be of great uncertainty and only qualitative identification is possible.

Assuming a well-defined background, the uncertainty in *S*_1_ and *S*_2_ due to the Poisson counting statistics will be:

(20) $$\sigma \left(S_{1}\right)\;=\sqrt{S_{1}+480}$$

(21) $$\sigma \left(S_{2}\right)\;=\sqrt{S_{2}+960}$$

**Figure 10:**
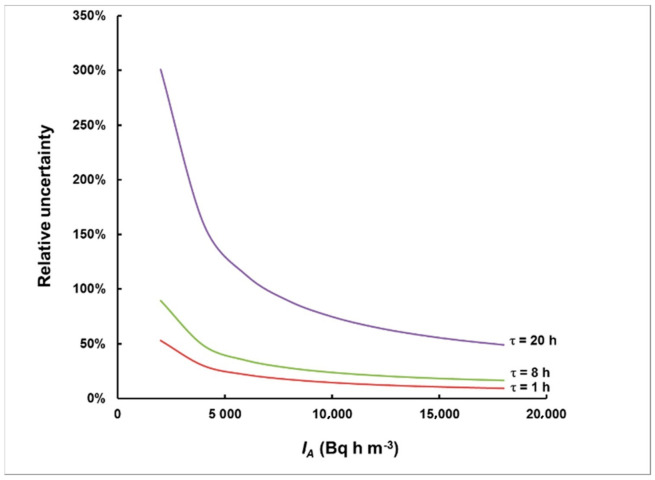
Relative uncertainty in the integrated activity concentration for measurements started with a delay of 1 h, 8 h and 20 h.

We have modeled uncertainty propagation, assuming Poisson distribution of the total number of counts in the ROI for the corresponding time interval. The results are illustrated in Figure 10.

As can be seen in Figure 10, the relative uncertainty is substantially influenced by the delay time. For a delay time of 20 h, only levels greater than 20,000 Bq h m^−3^ can be measured within 50% relative uncertainty, while, for a delay time of 8 h, 50% relative uncertainty could be achieved even for levels of approximately 5000 Bq h m^−3^. The delay time of 1 h makes it possible for levels >10,000 Bq h m^−3^ to be evaluated with a relative uncertainty better than 10%.

### 3.3. Simulation of Plumes for an Arbitrary Shape

**Figure 11:**
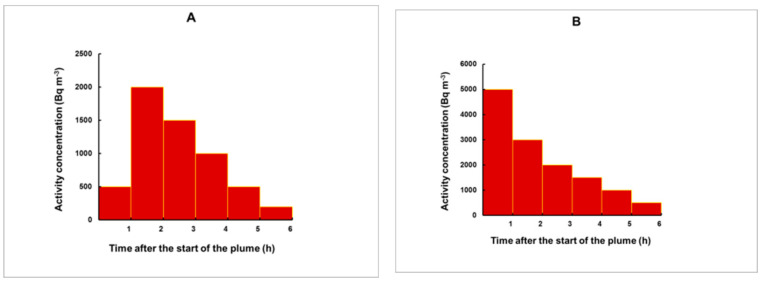

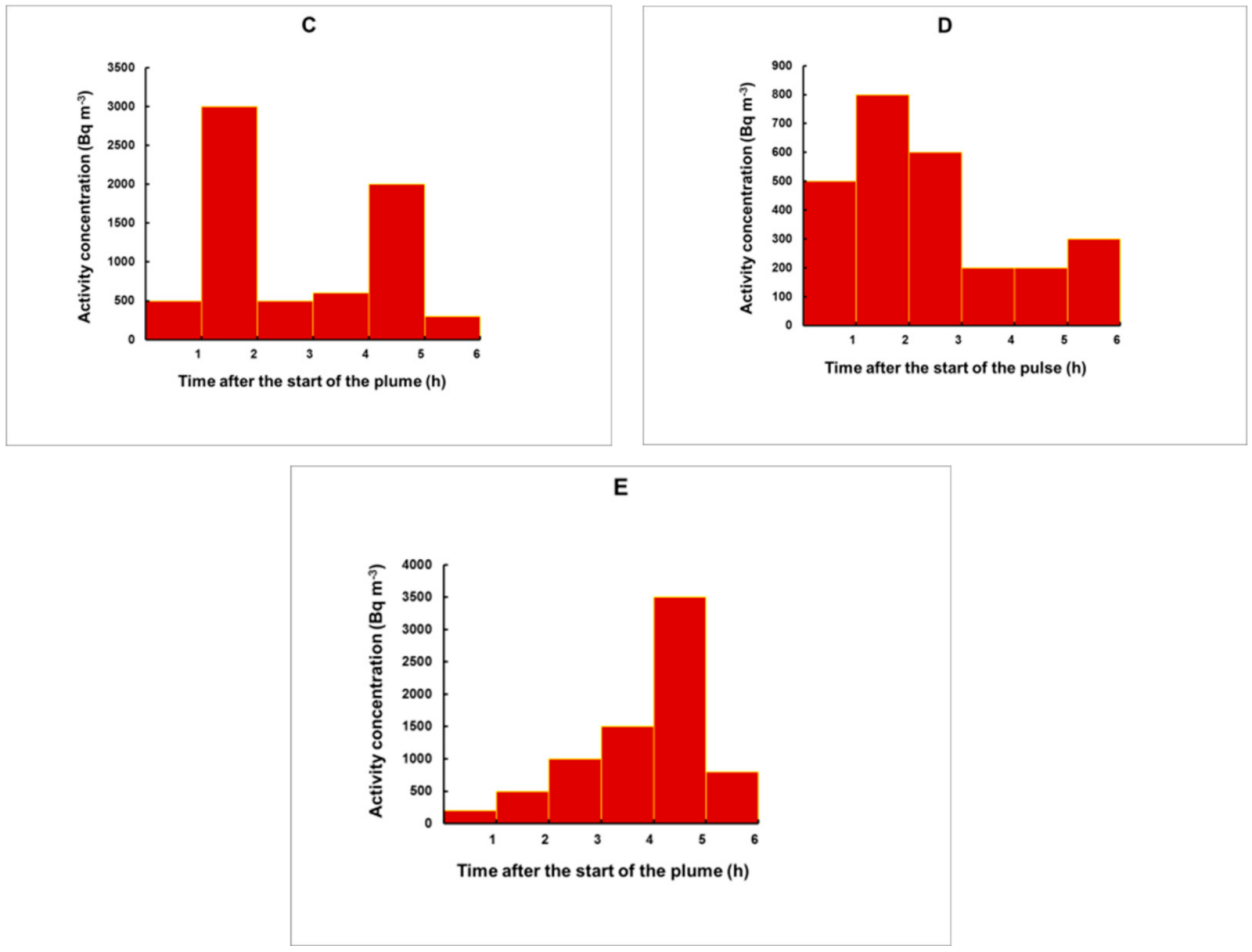
Five different plumes used to test the method. The plumes are composed of consecutive rectangular pulses, each with a duration of 1 h. Different profiles were modelled: Fast (**A**) and sharp (**B**) increase followed by a slow decrease; profile with two pulses of higher release (**C**); relatively weak plume with a release mostly during the first half of the plume (**D**); a plume with slowly increasing concentrations with a maximum close to its end (**E**).

To check whether the method may work reliably for pulses with irregular shape, we simulated different plume profiles, illustrated in Figure 11.

**Figure 12:**
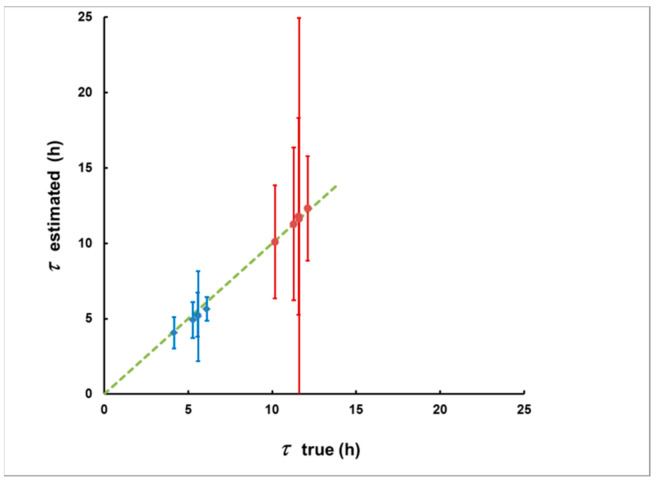
The estimated delays and their uncertainties due to the “counting statistics” (error bars) for the five plumes from Figure 11 and with the two delay scenarios (the first is in blue, the second is in red). Notably, the methodological bias (the deviation of the points from the dashed line that corresponds to the coincidence of the estimated and true delay) is much smaller than the statistical uncertainty.

**Figure 13:**
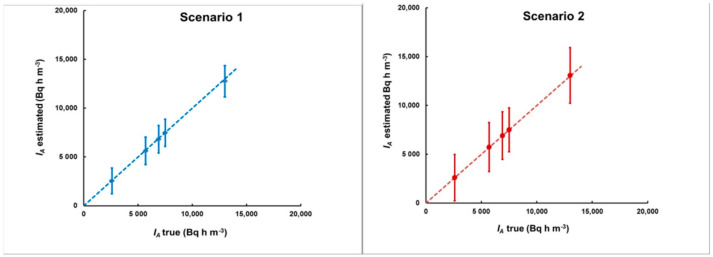
The estimated integrated activity concentrations for the plumes of Figure 11 for the first (**left**) and the second (**right**) delay scenario. As with the delay estimates, here, the methodological bias (the deviation of the points from the dashed line that corresponds to “estimated = true *I_A_*”) is also much smaller than the “counting statistics uncertainty” (error bars).

With each plume, two “measurement delay scenarios” were considered: the first one commencing 2 h after the end of the plume and a second one commencing 8 h after the end of the plume. Because the plumes were of different shape, the delays from the plume’s center were different. The delay was calculated by Equation (15) and the integrated activity by Equation (14). The results are illustrated in Figure 12 and Figure 13.

As can be seen from Figure 12 and Figure 13, in this case, the method also provides acceptable agreement between the estimated and “true” values. Therefore, we may conclude that the method can be applied for plumes of any shape.

## 4. Discussion and Conclusions

The present work demonstrates, by computer modeling, that, in polycarbonate specimens composed of Makrofol N foils of thickness 40 μm and 120 μm, exposed for a certain time interval in which a short-term ^133^Xe plume occurs, and with consecutive HPGe gamma spectrometry measurement in two time intervals, the following plume characteristics can be determined:

- The time (before the start of the gamma spectrometry measurement) at which the center of the plume was situated;
- The ^133^Xe activity concentration, integrated over the total duration of the plume, provided that the plume ended before the specimen was taken from the place of exposure. Otherwise, *I_A_* will refer to the time from the start of the plume until the moment when the specimen was removed for analysis.

By computer simulation of real plumes of rectangular and arbitrary shape, it was demonstrated (Figure 9) that even plumes of *I_A_* < 500 Bq h m^−3^ may be identified at 95% statistical significance if the measurement starts within the first 8 h after the moment of the plume center. Notably, this estimate was achieved for a gamma spectrometer of average class. The current state-of-the-art in the field of gamma spectrometry includes detectors of much better efficiency and detection systems with better suppressed background than these for the instrument whose characteristics were used. Therefore, one may anticipate that this method has the potential for better sensitivity than that estimated in the current study. Moreover, the choice of foil thickness and counting intervals in the present work was arbitrary, aiming to evaluate the feasibility of the method. Further research may model different foils and counting intervals, aiming for the optimization of the method.

The method provides data that can be useful for the evaluation of other characteristics of the release, e.g., the total released activity. For instance, if the specimen is placed in the ventilation stack through which the activity is released, to obtain the total released activity, one simply has to multiply the determined integrated activity concentration by the air flow rate through the stack. If the specimen is exposed to the environment, the use of an atmospheric transport model along with the data from the measurement will be needed. Thus far, estimates of the released activity, especially after accidents, are associated with great uncertainties and, sometimes, only the order of magnitude of the release may be assessed. In contrast, the modeling revealed that this method may provide *I_A_* estimates of uncertainty that may be potentially less than 10% (see Figure 10). Therefore, the proposed method has the potential to facilitate a step forward in this direction.

The absorption ability of polycarbonates is not affected by most of environmental factors (pressure, fume, dust, humidity within 0–100% and even wetting of the material [33,34]). The temperature is the only environmental factor of influence, which, however, may be taken into account, provided that the temperature dependences of *K* and *D* for xenon in Makrofol N are well-known. Currently, there are data available only for room temperature (22 °C) and the current modeling is based on these data. Dedicated research to complement the data set with values for other temperatures is planned for the future.

One important challenge is to test the method experimentally. Experimental tests with ^133^Xe are hampered by the difficult and expensive access to certified ^133^Xe sources and the difficulty of creating a reference ^133^Xe concentration that can be precisely controlled. We plan in the future to perform such experiments using, as a surrogate of ^133^Xe, the isotope ^222^Rn, for which such tests will be much easier. Of course, this will need revision of the method to incorporate data related to ^222^Rn for the parameters used.

The polycarbonate material is cheap and offers a cost-efficient option to place many specimens for a long time at different locations in the environment, as well as within a controlled facility, and to use them on occasion, or periodically for regular control. Such a network of specimens may be useful after real or suspected radioxenon release from a nuclear or radiopharmaceutical facility, or to check for possible clandestine nuclear activity. The measured specimens may provide data that will complement the data from stationary monitors and may be used to refine the atmospheric transport models of the plumes. In addition, after outgazing for 10 days or more, the specimen will be suitable for use again. The analysis of the specimens may be conducted in laboratories that are far from the site of exposure, which may be very useful in the event of a nuclear emergency, where the efforts to clarify the situation become international.

## Data Availability

The study did not report any data.
